# Loss of flotillin expression results in weakened desmosomal adhesion and *Pemphigus vulgaris*-like localisation of desmoglein-3 in human keratinocytes

**DOI:** 10.1038/srep28820

**Published:** 2016-06-27

**Authors:** Frauke Völlner, Jawahir Ali, Nina Kurrle, Yvonne Exner, Rüdiger Eming, Michael Hertl, Antje Banning, Ritva Tikkanen

**Affiliations:** 1 Institute of Biochemistry, Medical Faculty, University of Giessen, Friedrichstrasse 24, 35392 Giessen, Germany; 2 Department of Dermatology and Allergology, Philipps University of Marburg, Baldingerstrasse, 35043 Marburg, Germany

## Abstract

Desmosomes are adhesion plaques that mediate cell-cell adhesion in many tissues, including the epidermis, and generate mechanical resistance to tissues. The extracellular domains of desmosomal cadherin proteins, desmogleins and desmocollins, are required for the interaction with cadherins of the neighbouring cells, whereas their cytoplasmic tails associate with cytoplasmic proteins which mediate connection to intermediate filaments. Disruption of desmosomal adhesion by mutations, autoantibodies or bacterial toxins results in severe human disorders of e.g. the skin and the heart. Despite the vital role of desmosomes in various tissues, the details of their molecular assembly are not clear. We here show that the two members of the flotillin protein family directly interact with the cytoplasmic tails of desmogleins. Depletion of flotillins in human keratinocytes results in weakened desmosomal adhesion and reduced expression of desmoglein-3, most likely due to a reduction in the desmosomal pool due to increased turnover. In the absence of flotillins, desmoglein-3 shows an altered localisation pattern in the cell-cell junctions of keratinocytes, which is highly similar to the localisation observed upon treatment with *pemphigus vulgaris* autoantibodies. Thus, our data show that flotillins, which have previously been connected to the classical cadherins, are also of importance for the desmosomal cell adhesion.

Desmosomes are cell-cell adhesion structures that are characterised by the presence of desmosomal cadherins (DSM-Cad), the desmogleins (Dsg1–4) and desmocollins (Dsc1–3), and are important for the integrity of various tissues such as skin, heart and gut epithelium (Reviewed in^1^,^2^). Desmosomes are firmly coupled to the intermediate filament cytoskeleton, e.g. keratin filaments in epithelial tissues, which creates resistance to mechanical forces that the tissues are exposed to. The cytoplasmic tails of DSM-Cads are associated with proteins of the dense desmosomal plaque, such as the plakophilins and plakoglobin (also known as γ-catenin) that belong to the armadillo repeat protein family. The association with the cytoskeleton is mainly mediated by the desmoplakin proteins. The extracellular domains (ECs) of DSM-Cads contain cadherin-like repeats that mediate cell-cell adhesion by means of interactions with the DSM-Cads in the adjacent cells. DSM-Cad ECs are homologous to that of the classical E-cadherin that is found in adherens junctions (AJs), and desmosomal adhesion is dependent on extracellular calcium ions, similarly to AJ mediated adhesion.

Epithelial tissues usually express several isoforms of DSM-Cads. Whereas Dsg2 and Dsc2 are found in all cells containing desmosomes^3^,^4^, other DSM-Cads show a more restricted expression pattern. Dsg1, Dsg3, Dsc1 and Dsc3 are mainly expressed in stratified, squamous epithelial tissues such as the epidermis, where they exhibit differential expression patterns in the layers of the epidermis. The expression of Dsg1 and Dsc1 is highest in the upper layers of the epidermis, whereas Dsg3 and Dsc3 are abundant in the basal and suprabasal layers^5^,^6^.

Although desmosomal adhesion is very firm, consistent with the role of desmosomes in tissue integrity, desmosomes also undergo dynamic remodelling during processes like differentiation and wound healing (Reviewed in^7^). Thus, the balance between desmosome assembly and disassembly needs to be regulated during desmosome remodelling. Assembled desmosomes are highly insoluble structures also due to their association with the cytoskeleton. Before arrival to the plasma membrane and assembly into desmosomes, DSM-Cads are detergent soluble but become insoluble upon desmosomal assembly^8^. Thus, regulation of the soluble vs. insoluble pools of DSM-Cads also affects the strength of desmosomal adhesion. However, desmosomal adhesion is also controlled by means of uptake of DSM-Cads from the plasma membrane^7^.

Desmosomal adhesion is important for the integrity of the epidermis, as evidenced by human diseases that result from the weakening/loss of desmosomal adhesion. In *pemphigus vulgaris* (PV), IgG autoantibodies against Dsg3 cause a profound blistering of the skin and mucosal epithelia, whereas in *pemphigus foliaceus*, IgG autoantibodies against Dsg1 cause a blistering phenotype restricted to the skin (for a review, see^9^,^10^). Several molecular mechanisms including kinase signalling, steric hindrance and Dsg depletion by means of endocytosis apparently contribute to the blistering and dissolution of desmosomal adhesion (reviewed in^11^,^12^).

Flotillin-1 and -2 are ubiquitous proteins that are associated with lipid microdomains called membrane rafts. Flotillins are present in various cellular compartments, including the plasma membrane and endosomes, and they have been suggested to function in cellular signalling and membrane trafficking processes, including endocytosis and endosomal trafficking^13^. Recent findings have connected flotillins to cell-cell adhesion (reviewed in^14^), and various cell adhesion proteins, including E-cadherin, β-catenin and p120catenin have been shown to associate with flotillin microdomains in a cell type dependent manner^15^,^16^,^17^,^18^,^19^,^20^. Most of these findings have concentrated on proteins associated with AJs, but also DSM-Cads have been suggested to localise in flotillin dependent rafts^21^. We have recently shown that flotillins directly interact with plakoglobin which is localised in both AJs and desmosomes^16^. However, in MCF10A breast epithelial cells, flotillin-1 depletion did not alter the raft association of E-cadherin and plakoglobin, whereas flotillin-2 ablation even increased their presence in rafts^16^. On the other hand, in HT-29 colon carcinoma cells, E-cadherin and p120ctn associate with membrane rafts that are defined by flotillin-1^15^, indicating that flotillins may exhibit a cell type specific function in cell-cell adhesion.

Since plakoglobin is a direct interaction partner of both flotillins and DSM-Cads, it is plausible that flotillins might also play a role in desmosomal adhesion in epithelial tissues. This study was carried out to characterise the role of flotillins in desmosomal adhesion in the epidermis. We here show that flotillins partially colocalise and directly interact with the cytoplasmic tails of Dsg1–3. Flotillin ablation decreases the expression of Dsg3 in the human keratinocyte cell line HaCaT due to increased Dsg3 lysosomal turnover. Flotillin depletion impairs desmosomal adhesion strength and results in a localization of Dsg3 that is highly similar to that observed upon treatment of keratinocytes with PV antibodies. Desmosomal morphology in flotillin knockdown cells can be improved upon inhibition of dynamin-dependent endocytosis. Thus, flotillins are required for the integrity of desmosomal adhesion and for the proper expression of Dsg3 in epidermal keratinocytes. We postulate that flotillin depletion results in increased endocytosis and turnover and thus reduced expression of Dsg3. Therefore, flotillins appear to be important for the stabilisation of Dsg3 at the plasma membrane, and may thus also play a role in diseases such as PV.

## Results

### Flotillins colocalise and interact with Dsg3

**Figure 1:**
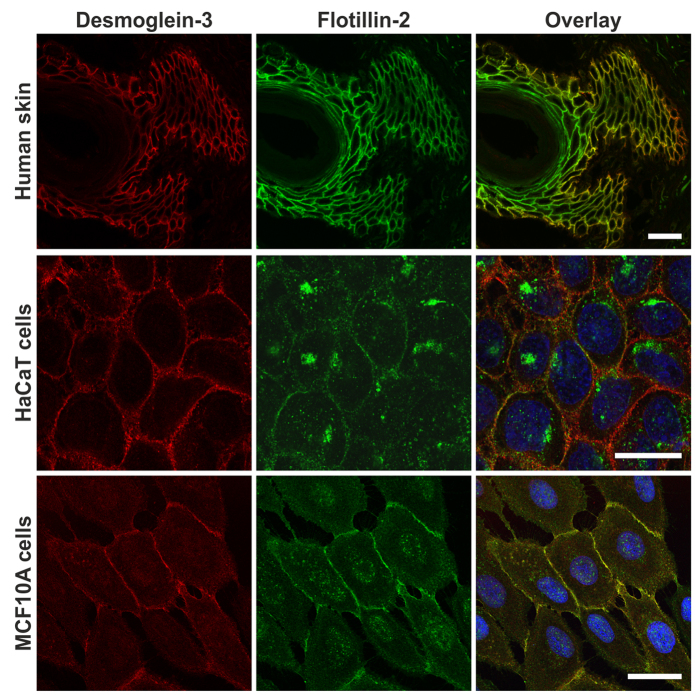
Desmoglein-3 and flotillin-2 colocalise in human epidermis and human epithelial cell lines. Human skin cryosections, HaCaT and MCF10A cells were immunostained for Dsg3 (red) and flotillin-2 (green). In human epidermis, flotillin-2 is expressed throughout the epidermal layer and colocalises with Dsg3 in keratinocytes of the stratum basale and suprabasale. In HaCaT keratinocytes, flotillin-2 is localised at the cell membrane and in intracellular vesicular structures. Dsg3 is localised at the plasma membrane where it colocalises with flotillin-2. In MCF10A mammary epithelial cells, flotillin-2 and Dsg3 show a more profound colocalisation at the sites of cell-cell contact. The cell lines were grown on glass coverslips for at least 3 days. Scale bar: 20 μm.

Our earlier data have shown that flotillins interact with γ-catenin which has been shown to localise in both AJs and desmosomes. Although the functional association of flotillins with E-cadherin and AJs is meanwhile well documented^15^,^16^,^17^,^18^,^19^,^20^, there are no direct data on a similar connection with desmosomes. Thus, the purpose of the present study was to characterise if flotillins play a role in desmosomal cell adhesion in the skin. Human skin cryosections were stained with antibodies against Dsg3 and flotillin-2 or flotillin-1, which were detected with fluorochrome labelled secondary antibodies (Fig. 1, uppermost row, and Supplementary Figure S1). Dsg3 was mainly localised in the basal/suprabasal layers of the epidermis, whereas flotillins showed a broader distribution that also reached the upper layer of the epidermis. However, in the basal/suprabasal layer, flotillins and Dsg3 colocalised at the cell-cell borders in the same cells. In the human HaCaT keratinocyte-like cell line^22^, Dsg3 and flotillin-2 showed a partial colocalisation at the plasma membrane (Fig. 1, middle row). However, a large fraction of flotillin-2 was localised in intracellular structures which did not contain Dsg3. In human MCF10A breast epithelial cells, in which flotillin-2 mainly resides at the plasma membrane, Dsg3 and flotillin-2 showed a more prominent colocalisation (Fig. 1, lowermost row).

**Figure 2:**
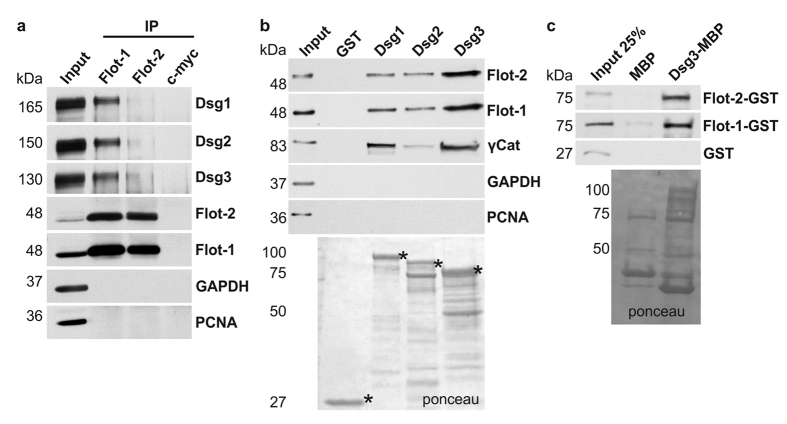
Flotillins directly interact with the cytoplasmic domain of desmogleins. (**a**) Co-immunoprecipitation of desmogleins 1–3 from HaCaT cell lysates with antibodies against endogenous flotillin-1 or flotillin-2. HaCaT cells were grown confluent for several days, and 1 mg of lysate was used for each experiment. Anti-c-myc was used as a negative control. (**b**) GST pulldown from HaCaT cell lysates using GST-tagged, purified proteins of the cytoplasmic domains of Dsg1–3. GST was used as a negative control. For each pulldown, 1 mg of cell lysate and 5 μg of purified protein was used. (**c**) Direct pulldown with purified recombinant proteins (Dsg3-MBP and Flot-GST). For each pulldown, 5 μg Dsg3-MBP or MBP were used and incubated with the indicated GST fusion proteins. Interaction of proteins was verified by Western blot.

Since the resolution of confocal microscopy is not sufficient to conclude that two proteins that colocalise are truly associated with each other as a complex, a possible interaction of flotillins with desmogleins 1, 2 and 3 was tested using biochemical means. When flotillin-1 or -2 was immunoprecipitated from HaCaT cells, a fraction of Dsg1–3 was found to coprecipitate, especially with flotillin-1 (Fig. 2a). When the cytoplasmic tails of Dsg1, -2 or -3 were expressed as GST fusion proteins and used in a pulldown assay, flotillin-1 and -2 were pulled down from HaCaT lysates (Fig. 2b). The relative amount (normalised to the amount of the fusion protein) of flotillins pulled down by Dsg3 tail was about twice as high as the amount detected in Dsg1 and Dsg2 pulldowns. The interaction between Dsg3 and flotillins was shown to be direct, since recombinantly expressed, purified flotillin-GST fusions readily interacted with purified Dsg3-MBP fusion protein (Fig. 2c).

### Flotillin depletion reduces the expression of Dsg3 in keratinocytes

**Figure 3:**
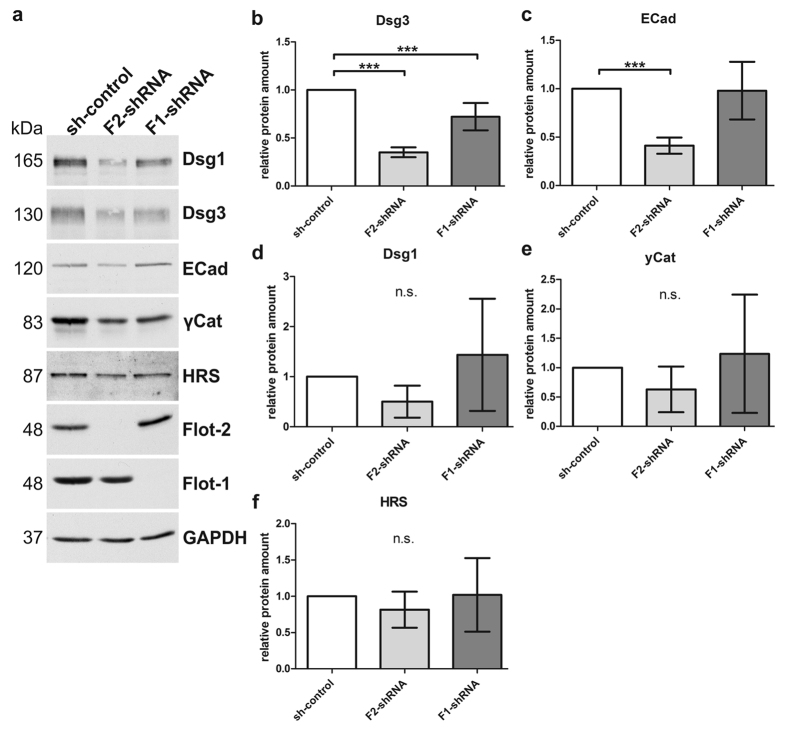
Flotillin depletion results in diminished Dsg3 and E-cadherin protein levels in HaCaT cells. (**a**) HaCaT cells with a stable flotillin knockdown or control cells were grown until confluent, and equal protein amounts were analysed by Western blot. (**b–f**) Western blot signals were quantified by scanning densitometry and normalised against GAPDH. Bars represent the mean ± SD of five (**b–e**) or four (**f**) independent experiments. One-way ANOVA with Bonferroni’s multiple comparison test. ***p < 0.001.

We have earlier shown that stable, shRNA mediated knockdown of flotillins in MCF10A cells does not result in changes in the expression of E-cadherin or α-, β- or γ-catenin^16^. However, in stable flotillin knockdown HaCaT cells, the expression of some adhesion proteins was reduced (Fig. 3a). Importantly, Dsg3 expression was significantly reduced upon knockdown of either flotillin-1 or flotillin-2 (Fig. 3b), whereas E-cadherin expression was significantly diminished only in flotillin-2 knockdown cells (Fig. 3c). Dsg1 and γ-catenin showed a highly variable expression upon loss of flotillin-1, but neither Dsg1 nor γ-catenin was significantly altered in flotillin knockdown cells (Fig. 3d,e). Expression of an unrelated control protein, Hrs (hepatocyte growth factor-regulated tyrosine kinase substrate), was also not significantly altered upon flotillin knockdown (Fig. 3f). Thus, flotillin expression appears to be important for the stability of specific adhesion proteins in human HaCaT keratinocytes.

**Figure 4:**
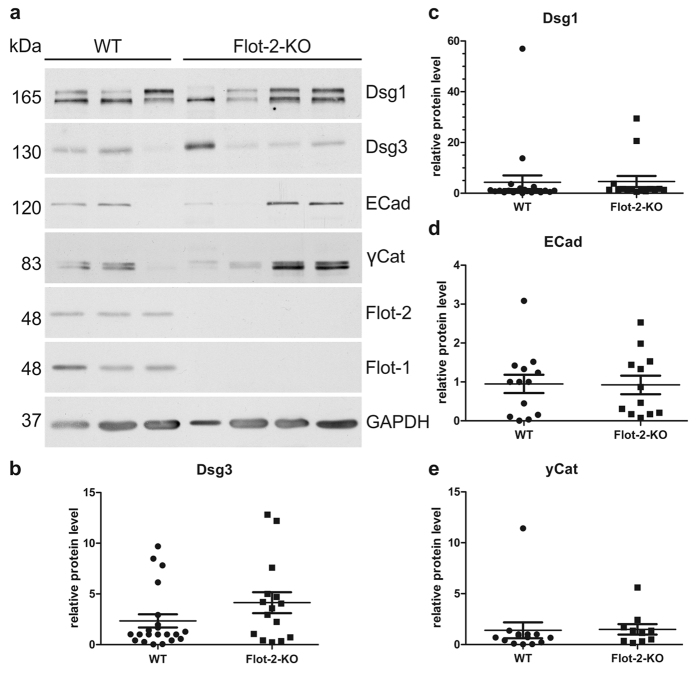
Expression of adhesion proteins in flotillin-2 knockout mouse skin. (**a**) Skin of adult wildtype (WT) and flotillin-2 knockout (Flot-2-KO) mice was lysed, and the levels of adhesion proteins were analysed by Western blot. (**b–e**) Western blot signals for Dsg3, Dsg1, E-cadherin and γ-catenin were quantified by scanning densitometry and normalised against GAPDH. Each data point represents the relative protein amount in a mouse skin lysate. A randomly chosen wildtype sample was set as 1, and all other samples were normalised to it. The mean ± SD is indicated for each protein. Number of analysed animals: (**b**) WT: 21, Flot-2-KO: 15. (**c**) WT: 21, Flot-2-KO: 15. (**d**) WT: 13, Flot-2-KO: 12. e) WT: 14, Flot-2-KO: 10.

To verify these findings in a more physiological context, the expression of adhesion proteins was analysed in the epidermis of flotillin-2 knockout mice that we have recently generated^23^ (Fig. 4a). Dsg3 showed a tendency to be upregulated, but the data failed to become significant due to the generally large variation of Dsg3 expression between different mice (Fig. 4b). However, the expression of Dsg1, E-cadherin and γ-catenin was not changed upon flotillin-2 knockout (Fig. 4c–e). We also performed immunostaining of control and flotillin-2 knockout mouse skin for desmoplakin and Dsg1 (Supplementary Figure S2), but no major changes in their staining pattern was detected upon flotillin-2 ablation. Staining for Dsg3 was not possible, despite our efforts, since the monoclonal antibodies gave too much background staining in the mouse skin sections.

### Flotillin depletion results in weakened desmosomal adhesion

**Figure 5:**
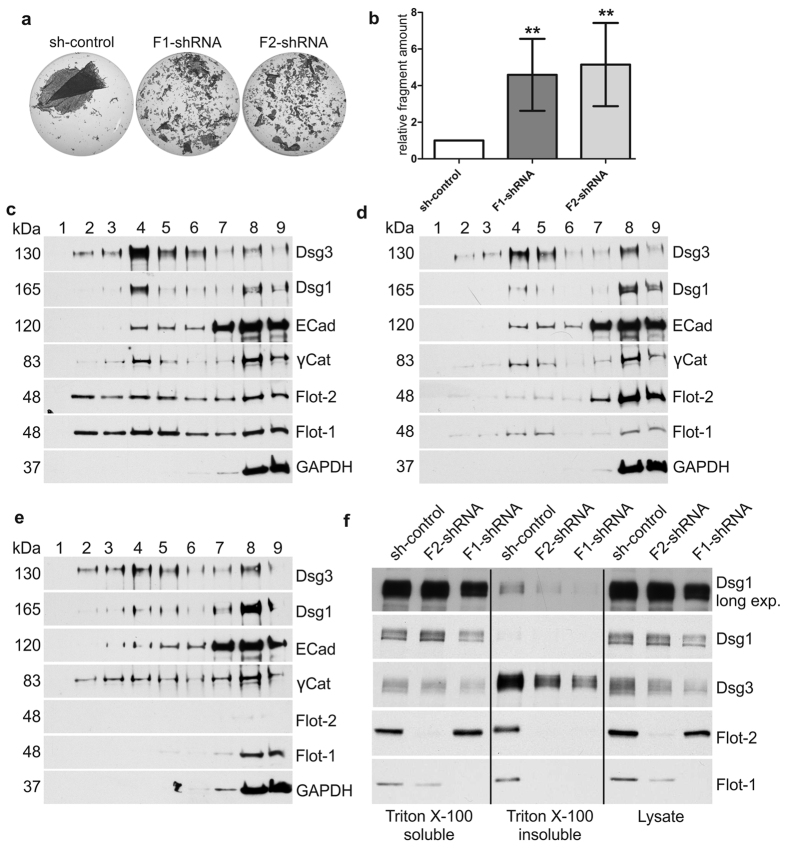
Flotillin knockdown weakens the adhesion strength in HaCaT keratinocytes but does not affect the raft association or detergent solubility of Dsg1 and Dsg3. (**a**) HaCaT cells depleted of flotillin-1 (F1-shRNA) and flotillin-2 (F2-shRNA) and control shRNA cells (sh-control) were dissociated from the plate with dispase and the monolayers were mechanically disrupted. Fragments were fixed, stained and imaged. (**b**) Relative fragment number from six independent experiments. Bars represent the mean ± SD. One-way ANOVA with Bonferroni’s multiple comparison test. *p < 0,05, **p < 0,01, ***p < 0.001. (**c–e**) Rafts were isolated from control shRNA (**c**) and flotillin-1 (**d**) or flotillin-2 (**e**) depleted HaCaT cells using detergent extraction and density gradient centrifugation. Nine fractions were collected from the top of the gradient (fraction 1 = the lightest fraction) to the bottom and analysed by Western blot. (**f**) Sequential detergent extraction of HaCaT control and flotillin knockdown cells. Cells were initially extracted with a Triton X-100 buffer followed by solubilisation of the Triton X-100 insoluble proteins with an SDS/urea buffer. Equal volumes of extracted proteins were analysed by Western blot. Please note that the total volume of the insoluble pool is twice that of the soluble one. The lysates shown in the last three rows were extracted with a NP-40 containing lysis buffer to facilitate solubilisation. The quantifications for (**c–f**) are shown in Supplementary Figure S3.

A change in the expression of adhesion proteins may result in concomitant changes in the adhesiveness mediated by the respective adhesion structures. An assay based on mechanical dissociation of a floating cell monolayer can measure the relative strength of desmosome mediated cell adhesion^24^. Confluent control and flotillin knockdown HaCaT keratinocytes were treated with dispase to dissociate the monolayers from the culture dish. After that, the cells were dispersed by repeated pipetting, and the resulting fragments were quantified (Fig. 5a). Flotillin-1 or -2 knockdown cells exhibited significantly more fragments than control shRNA cells (Fig. 5b). These results show that desmosomal adhesion in HaCaT keratinocytes is weakened upon flotillin depletion.

### Flotillin depletion does not change raft association of Dsg3 and Dsg1 but reduces the absolute amount of detergent-insoluble Dsg3

Several desmosomal proteins, including Dsg3, have been found to be associated with detergent-insoluble membrane domains or membrane rafts^25^,^26^. However, detergent insolubility of desmosomal proteins is also mediated by their strong association with the cytoskeleton (keratin filaments), which is not equal to raft association. To measure both raft association and plain detergent insolubility due to cytoskeletal association, two assays were used. Association of desmosomal proteins with membrane rafts was studied using detergent extraction and density gradient centrifugation, whereas general detergent insolubility was monitored after detergent extraction only. The latter assay is generally used to monitor the desmosomal (insoluble, cytoskeleton associated) and extradesmosomal (detergent soluble) membrane pools of desmogleins^27^,^28^. The distribution in density gradients and thus membrane raft association of Dsg3, Dsg1 and γ-catenin was not significantly altered in flotillin-knockdown HaCaT cells (Fig. 5c–e, quantification in Supplementary Figure S3). A major part of Dsg3 was found to be raft localised, whereas a minor fraction of Dsg1, E-cadherin or γ-catenin was found in the raft fractions 1–5. These data are also consistent with previous findings that although depletion of one flotillin impairs the raft association of the other one (Fig. 5c–e), rafts are not generally impaired^16^,^29^, and the raft association of desmosomal proteins apparently remains intact.

When cell fractionation after detergent extraction was performed, the relative detergent insolubility of Dsg3 was not changed upon flotillin knockdown (Fig. 5f, quantification in Supplementary Figure S3). However, a much smaller total amount of Dsg3 was found in the detergent-insoluble, desmosomal fraction as compared to control cells, due to the reduction of the total Dsg3 expression levels in flotillin knockdown cells. Consistent with the data shown in Fig. 5c–e, only a minor fraction of Dsg1 was found to be detergent-insoluble in control and flotillin knockdown cells (Fig. 5f). Thus, these data might imply that desmosomal adhesion is weakened upon loss of flotillins due to a lower total amount of Dsg3 in desmosomes, whereas the relative detergent insolubility and raft association of Dsg3 is not changed.

### Localisation of Dsg3 is altered in flotillin knockdown keratinocytes

**Figure 6:**
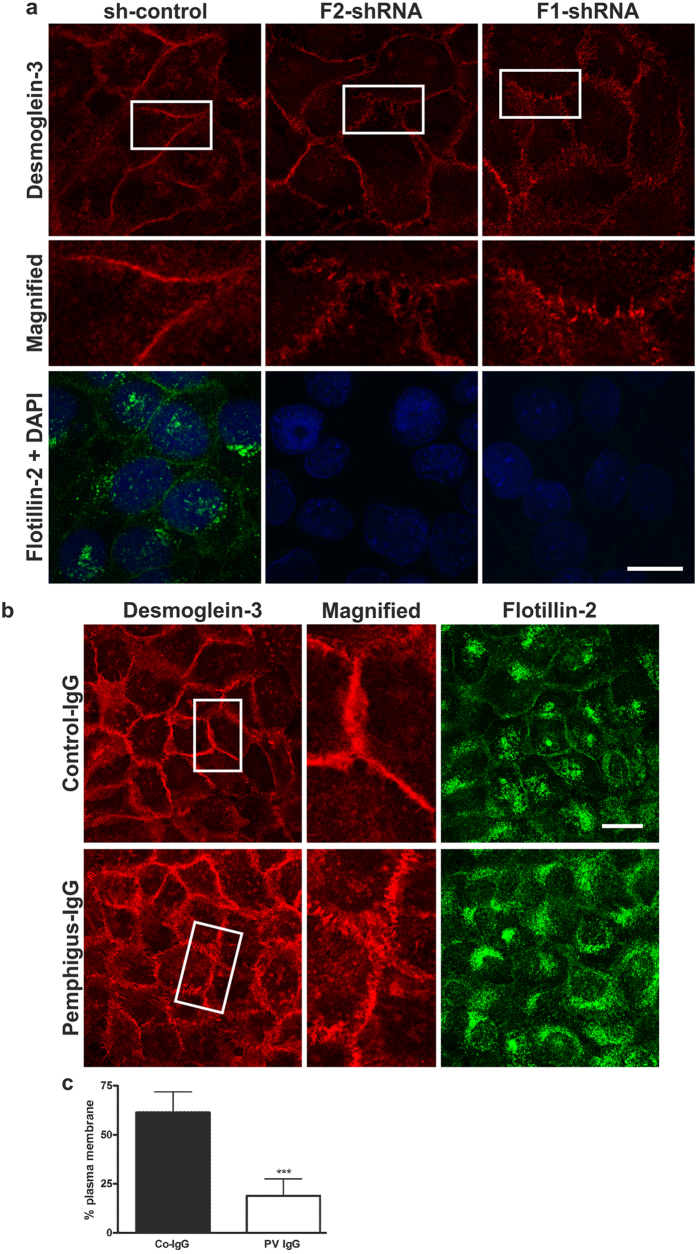
Loss of flotillins results in *pemphigus vulgaris* like localisation of Dsg3 in HaCaT keratinocytes. (**a**) HaCaT cells depleted of flotillin-1 (F1-shRNA), flotillin-2 (F2-shRNA) or control shRNA cells (sh-control) were grown for three days on glass coverslips, fixed and stained with Dsg3 (red) and flotillin-2 (green) antibodies. Note that the signals for Dsg3 in flotillin knockdown cells in (**a**) have been adjusted to make the morphological deficiencies more clear. (**b**) HaCaT keratinocytes were grown for two days on glass coverslips and treated with 150 μg/ml of either control-IgG (healthy person) or IgG from PV patients for 20 h. Cells were fixed and immunostained for Dsg3 (red) and flotillin-2 (green). Scale bar: 20 μm. (**c**) Quantification of flotillin-2 localisation in control IgG vs. PV IgG treated cells. At least 8 images/condition (total of >200 cells/condition) originating from three independent experiments were quantified for plasma membrane associated vs. intracellular flotillin-2 staining. Statistical analysis was done by Student’s t test (***p < 0.001).

To test if flotillin knockdown displays an effect on the localisation of Dsg3 or on the morphology of desmosomes, Dsg3 was immunostained in confluent HaCaT cells (Fig. 6a). In control shRNA cells, Dsg3 showed a sharp, continuous localisation at the cell borders (Fig. 6a, left). However, in flotillin-1 and -2 knockdown cells, Dsg3 showed a more disorganized localisation in punctate/linear structures (Fig. 6a, middle and right). Please note that the Dsg3 signal intensity in flotillin knockdown cells was adjusted in order to compensate for the reduced expression and to make the aberrant localisation more clearly visible. Immunostainings for Dsg1, desmoplakin and γ-catenin demonstrated that the staining pattern of further desmosomal proteins was also altered upon flotillin knockdown (Supplementary Figure S4).

### Pemphigus vulgaris autoantibodies induce a change in flotillin localisation

*Pemphigus vulgaris* (PV) is a human autoimmune disease that is characterised by autoantibodies against Dsg3. These antibodies result in loss of desmosomal cell adhesion and acantholysis of epidermal keratinocytes^9^. When HaCaT keratinocytes were incubated with the IgG fraction isolated from the sera of PV patients, Dsg3 staining became more discontinuous and punctate/linear (Fig. 6b), exhibiting a staining pattern that resembled that in flotillin knockdown cells. Intriguingly, the PV IgG fraction also induced a redistribution of flotillin-2, and the compact intracellular staining observed in cells incubated with human control IgG became very diffuse and the plasma membrane staining was almost completely lost (Fig. 6b). Quantification of the data showed that whereas the total signals did not significantly change, a significantly reduced fraction of flotillin-2 was localised in the plasma membrane after PV IgG treatment (Fig. 6c). These data suggest that flotillins may be important for the integrity of desmosomes not only in healthy but also in PV skin, and that loss of flotillins from the plasma membrane may contribute to impaired desmosomal integrity upon PV IgG treatment.

**Figure 7:**
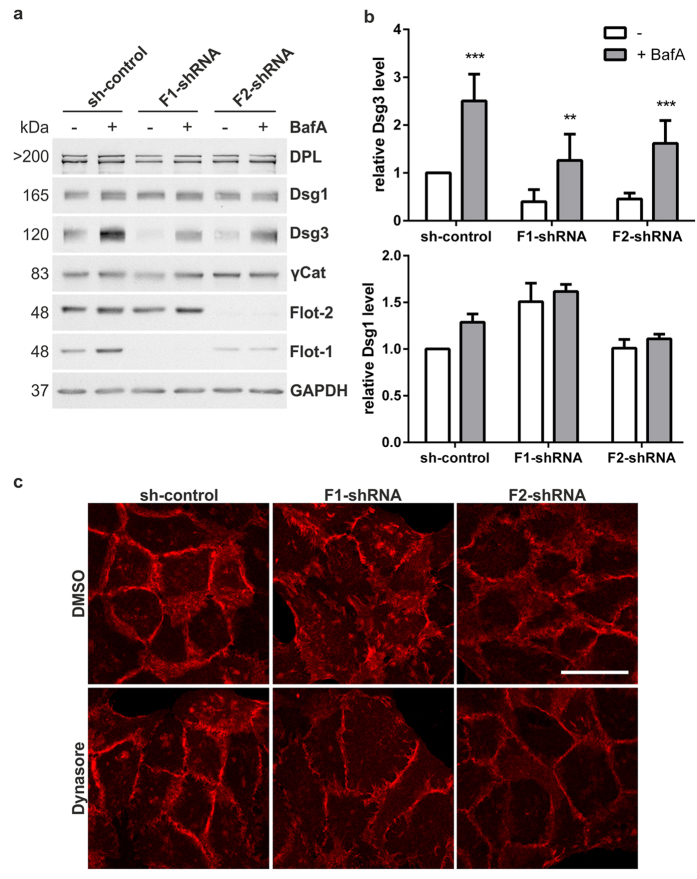
Inhibition of lysosomal degradation or endocytosis restores Dsg3 level and improves desmosomal morphology in flotillin knockdown cells. (**a**) Flotillin depleted or control HaCaT cells were treated for 24 h with BafA (50 nM) to block lysosomal degradation. Cell lysates were analysed by Western blot. (**b**) Western blot signals for Dsg3 (upper) and Dsg1 (lower) were quantified by scanning densitometry and normalised against GAPDH. Bars represent the mean ± SD of 6 independent experiments. Two-way ANOVA. *p < 0,05, **p < 0,01, ***p < 0.001. (**c**) Flotillin knockdown or control cells were serum-starved overnight and treated for two hours with 80 μM Dynasore to inhibit dynamin-dependent endocytic uptake of Dsg3 from the plasma membrane. DMSO served as a control. Cells were fixed and immunostained for Dsg3. Scale bar: 20 μm.

The reduced Dsg3 amount in flotillin knockdown cells might be due to increased endocytosis and lysosomal turnover of Dsg3. To specifically address this possibility, we treated the cells with Bafilomycin A (BafA), which increases lysosomal pH and thus inhibits lysosomal protein degradation (Fig. 7a). Quantitative assessment showed that BafA treatment significantly increased Dsg3 levels in flotillin knockdown and control cells, whereas Dsg1 (Fig. 7b), desmoplakin and γ-catenin levels did not significantly change (Supplementary Figure S5). Consistent with impaired lysosomal degradation upon BafA treatment, Dsg3 was found to heavily colocalise with LAMP1, a lysosomal marker, in BafA treated cells (Supplementary Figure S5). Altogether, these data suggest that the reduced amount of Dsg3 in the absence of flotillins may indeed be caused by its increased lysosomal turnover.

Dsg3 degradation in lysosomes requires it to be endocytosed from the plasma membrane, and endocytosis of Dsg3 in primary keratinocytes has been shown to be clathrin-independent but dependent on cholesterol^30^. Although Delva *et al*. also suggested that PV IgG-induced Dsg3 endocytosis is independent of dynamin, this was based only on overexpression of the dominant-negative dynamin-2 mutant Lys44Ala^30^, which is nowadays not considered to be sufficient to exclude dynamin dependency. Furthermore, endocytosis that regulates Dsg3 physiological turnover may well proceed through a different mechanism that requires dynamin. Thus, impairment of dynamin-dependent endocytosis may result in an increased amount of Dsg3 at the plasma membrane in flotillin knockdown cells, and possibly improvement of desmosomal morphology. To test this, control and flotillin knockdown cells were treated with Dynasore or MitMab, two chemical inhibitors of dynamin function. We have previously shown that these inhibitors also impair EGF-induced flotillin endocytosis^31^. A clear improvement of Dsg3 staining pattern in flotillin knockdown HaCaT cells was observed upon Dynasore (Fig. 7c) or MitMab (Supplementary Figure S6) treatment. These data suggest that when physiological Dsg3 endocytosis is inhibited and more Dsg3 becomes available at the plasma membrane, the impaired desmosomal morphology in flotillin knockdown cells can be ameliorated.

## Discussion

We have here shown that flotillins directly interact with the cytoplasmic tail of the desmoglein family members, and flotillin depletion in keratinocytes results in reduced expression and mislocalisation of Dsg3. Importantly, flotillin ablation also impairs the strength of desmosomal adhesion in keratinocytes, demonstrating that flotillins are novel factors that play a role in the integrity of cell-cell adhesion mediated by desmosomes. Previous studies have suggested a function for flotillins in the regulation of AJs^32^,^33^,^34^, whereas to our knowledge, they have not been functionally connected to desmosomal adhesion before. Since flotillins interact with both DSM-Cads, as shown here, and plakoglobin^16^, they are likely to be involved in processes such as assembly or disassembly of desmosomes.

Our data show that among the desmosomal proteins analysed, Dsg3 was the only one whose expression was significantly reduced upon flotillin depletion in HaCaT keratinocytes. However, Dsg1 and γ-catenin showed a tendency to be reduced in flotillin-2 knockdown cells, whereas in flotillin-1 knockdown cells, they exhibited a highly variable expression. The cause of this variation is at present unknown, as we have used highly confluent cells grown for several days for all experiments. Thus, it is possible that under certain circumstances, the expression of Dsg1 and γ-catenin may also be significantly reduced upon flotillin ablation. In the epidermis of our flotillin-2 knockout mice, the expression of Dsg3, Dsg1, E-cadherin and γ-catenin was not significantly changed. This may be due to the different organization of the cells (monolayer vs. multilayer), species difference (human vs. mouse), or caused by compensational effects upon loss of flotillin expression. We have earlier shown that our flotillin-2 knockout mice exhibit a profound transcriptional compensation that results in hyperactivity of signalling cascades such as the mitogen-activated protein kinase pathway, and in overexpression of downstream genes regulated by this pathway^23^. It is also important to note that both flotillin-2 and flotillin-1 expression are largely lost in our knockout mice^23^, whereas our HaCaT cell lines exhibit a knockdown of only one specific flotillin. Furthermore, our knockout mice have not yet been analysed under conditions of mechanical or biological stress, such as inflammation or exposure to PV IgG, in terms of a skin phenotype. In future experiments, we are planning to study the effect of such challenges on the epidermis of our knockout mice, since these experiments vexperiments may well reveal a hidden phenotype that is only seen under specific conditions.

Desmosomal assembly has been suggested to be a membrane raft mediated process, and several desmosomal components have been found to be associated with rafts^21^,^28^. Since flotillins form a specific raft microdomain which is different from e.g. caveolae, it would be plausible that flotillins might regulate the raft association of desmosomal proteins, and thus coordinate desmosomal assembly. However, we did not observe any significant changes in the relative raft association of Dsg3, Dsg1 or γ-catenin in our density gradient experiments, when measured as fraction (%) of the total protein. The same was true for the detergent extraction which reveals the insoluble (desmosomal) and soluble (non-desmosomal) pools. However, considering that Dsg3 expression is much lower in the flotillin knockdown cells, the absolute amount of Dsg3 was reduced in both the raft fractions and in the desmosomal pool. This is likely to result in weaker desmosomal adhesion in the absence of flotillins.

One potential function of flotillins in desmosomes could be the endocytosis of DSM-Cads, as flotillin-1 has been suggested to be a mechanistic part of a non-clathrin, non-caveolin endocytosis pathway that presumably operates via membrane rafts^35^,^36^, and endocytosis of Dsg2 and -3 has been shown to be clathrin-independent but dependent on cholesterol^30^,^37^. However, recent findings have shown that flotillin-1 is not required for the endocytosis of at least Dsg2, which is mediated by a cholesterol sensitive, dynamin-dependent pathway^37^. Furthermore, our data imply that Dsg3 turnover, which takes place by means of endocytosis and lysosomal degradation^30^,^38^, is increased in the absence of flotillins, resulting in reduced stability of desmosomal adhesion. However, PV IgG-mediated and physiological Dsg3 endocytosis may not proceed through identical routes, as our data here reveal a dynamin dependency for the physiological turnover, in contrast to the suggested non-dynamin endocytosis upon PV IgG treatment^30^.

Desmosomal morphology in flotillin knockdown cells could be improved upon inhibition of dynamin dependent endocytosis, further implicating that increased endocytosis and turnover of Dsg3 may be the major reason for weakened desmosomal adhesion upon loss of flotillins. Thus, our data suggest that flotillins may be required for the recruitment or stabilisation of Dsg3 in desmosomes. Previous studies have pointed to a role of membrane rafts in the stabilisation of desmosomal adhesion, and disruption of rafts by cholesterol perturbing agents results in loss of desmosomal adhesion^21^,^28^. However, it is not known which types of rafts regulate desmosomal adhesion in keratinocytes. In Madine-Darby canine kidney cells transfected with desmocollin-2-YFP, these rafts colocalise with ostreolysin, a mushroom protein that specifically binds to raft membranes^21^, but it is not clear which endogenous proteins are present in these rafts. Previous data on the role of rafts in desmosomal assembly are consistent with a model in which flotillins, and not rafts in general, may be required for the stabilisation of DSM-Cads in rafts, which would then facilitate the formation of firm desmosomes.

**Figure 8:**
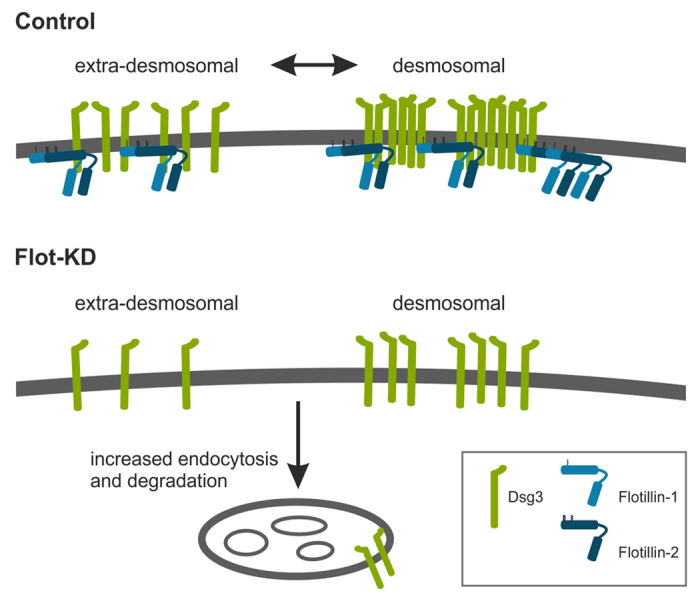
Schematic model for flotillin function in desmosomal adhesion in keratinocytes. Dsg3 (and other desmosomal cadherins) exist in two different plasma membrane-associated pools. The extra-desmosomal pool represents cadherins that are plasma membrane associated but not part of the mature desmosomes. It is assumed that lateral exchange takes place between both cadherin pools. We propose that flotillins are associated with Dsg3 plasma membrane pools and are required to stabilise Dsg3 at the plasma membrane. Absence of flotillins increases dynamin-dependent endocytosis and lysosomal degradation of Dsg3, which results in reduced total cellular amount of Dsg3 and weakens desmosomal adhesion.

Intriguingly, we could show that upon flotillin depletion, Dsg3 is mislocalised at the plasma membrane and exhibits a staining pattern with linear arrays that appear perpendicular to the plasma membrane. This pattern is highly similar to the localization of Dsg3 induced by the treatment of keratinocytes with PV IgG. Previous studies have shown that such linear arrays represent sites of Dsg endocytosis^28^,^39^. These findings would provide further support for a role of flotillins in the regulation of desmosome assembly and stabilisation of DSM-Cads at the plasma membrane. The observed mislocalisation of Dsg3 in flotillin knockdown cells is consistent with its reduced stability in desmosomes, enhanced localisation in sites of endocytosis and thus increased turnover by means of endocytosis, as shown by our data. In line with this, flotillins have been demonstrated to function as scaffolders of various membrane proteins in rafts (reviewed in^13^,^40^,^41^). Therefore, we propose a model (Fig. 8) according to which flotillins directly interact with desmosomal proteins, especially Dsgs, and scaffold them at the plasma membrane in clusters, which results in stabilisation of their plasma membrane localisation. A similar clustering function of flotillins has already been suggested by us and others during e.g. receptor signalling and uptake^29^,^42^. In the absence of flotillins, DSM-Cads may not be efficiently clustered and stabilised at the plasma membrane, resulting in increased endocytosis and degradation of Dsg3 (and possibly other desmosomal cadherins), and thus in weaker desmosomal adhesion. However, we currently do not know if flotillins interact with the desmosomal or non-desmosomal pools of desmogleins or with both. Since PV IgG treatment results in loss of plasma membrane staining of flotillins due to their uptake in intracellular compartments, this may also contribute to the destabilisation of desmosomal adhesion in *pemphigus vulgaris*. Therefore, it will be important in future studies to characterise the molecular details of the function of flotillin in desmosomal adhesion in keratinocytes, but also in other epithelial cells.

## Methods

### Plasmids

Full length rat flotillin-1 and flotillin-2 in pET41a vector have been described earlier^16^,^29^. Cytoplasmic domains of human desmogleins 1–3 were cloned by polymerase chain reaction from HaCaT cells into the pGEX-4T1 vector with glutathione-S-transferase (GST) tag or into the pMAL-c2X vector with maltose binding protein (MBP) tag. The PCR primers used to generate these constructs were as follows:

Dsg1: CTATAGGATCCTGTGATTGTGGAGGTGCTCCTC (fwd)

  CTATAGAATTCCTACTTGCTATATTGCACGGTAC (rev)

Dsg2: CTATAGGATCCATGTGCCATTGCGGAAAGGGC (fwd)

  CTATAGTCGACTTAGGAGTAAGAATGCTGTACAG (rev)

Dsg3: CTATAGAATTCTTGACCTGTGACTGTGGGGCAG (fwd)

  CTATAGTCGACTCATATTAGACGGGAGCAAGGATC (rev)

The Dsg3 variant corresponds to the reference sequence NM_001944.2^9^, whereas the Dsg2 natural variant Lys773Arg has been described^43^,^44^. All constructs were verified by sequencing.

### Antibodies

A mouse monoclonal antibody (BD Transduction Laboratories) was used to detect desmoglein-1 in Western blots. For immunofluorescence, a rabbit polyclonal antibody (Santa Cruz, Heidelberg, Germany) was used against Dsg1. Dsg2 was detected with a mouse monoclonal antibody (Santa Cruz). Dsg3 was detected with a mouse monoclonal antibody from AbD Serotec (Puchheim, Germany). A rabbit polyclonal antibody was used against desmoplakin I/II, and a mouse monoclonal antibody against PCNA (both from Santa Cruz). Mouse monoclonal antibodies against y-catenin, E-cadherin, flotillin-1 and flotillin-2 were purchased from BD Transduction Laboratories. The flotillin-1 antibody from BD was also used for the detection of flotillin-1 in the immunofluorescence shown in Supplementary Figure S1. A rabbit anti-E-cadherin antibody (Tebu-Bio, Offenbach, Germany) was used to detect E-cadherin in mouse skin lysates. To detect flotillins in mouse skin lysates, a rabbit anti-flotillin-2 antibody (Cell Signalling Technologies, Danvers, MA, USA) or a rabbit anti-flotillin-1 antibody (Sigma-Aldrich, Taufkirchen, Germany) were used. For immunofluorescence, a rabbit polyclonal antibody against flotillin-2 was used (Sigma-Aldrich). A mouse monoclonal antibody against GAPDH was obtained from Abcam (Cambridge, UK), and the polyclonal anti-Hrs antibody from Santa Cruz. A rabbit monoclonal antibody against c-myc (Santa Cruz, Heidelberg, Germany) was used as negative control for immunoprecipitation experiments. Secondary antibodies used in Western blots were horseradish peroxidase coupled goat anti-rabbit or goat anti-mouse antibodies obtained from Dako (Glostrup, Denmark). Secondary antibodies used for immunofluorescence were either a Cy3-conjugated goat anti-mouse antibody (Jackson ImmunoResearch, Newmarket, UK) or an Alexa Fluor 488-conjugated donkey anti-rabbit antibody (Life Technologies, Darmstadt, Germany).

### Cell culture and shRNA knockdown

The stable flotillin-1 and flotillin-2 knockdown HaCaT cells have been described in^45^. HaCaT cells were maintained in Dulbecco’s modified Eagle’s medium (DMEM) with high glucose and 10% foetal calf serum, 1% penicillin/streptomycin, 1% non-essential amino acids and 1% sodium pyruvate. Stable flotillin knockdown HaCaT cells were maintained in the same medium, supplemented with 2 μg/ml puromycin. MCF10A cells were cultured in DMEM Nutrient Mixture F-12, supplemented with 5% horse serum, 1% penicillin/streptomycin, 10 mg/ml insulin, 20 ng/ml human recombinant EGF (Sigma-Aldrich), 1 mM dexamethasone, 100 ng/ml cholera toxin (from *vibrio cholerae*). All cells were cultured at 37 °C under 8% CO_2_.

### Cell lysis, immunoprecipitation, gel electrophoresis and Western Blot

Cells were scraped into lysis buffer (50 mM Tris pH 7.4; 150 mM NaCl; 2 mM EDTA; 1% NP-40) supplemented with protease inhibitor cocktail and incubated on ice for 30 min. The cell lysate was cleared by centrifugation, and the protein amount was measured using Bradford assay. Equal protein concentrations were analysed by SDS-PAGE and Western blot. Immunoprecipitation from HaCaT cells was done as described earlier for MCF10A cells^16^, but 1 mg of protein was used.

### Flotillin-2 knockout mice and preparation of mouse skin lysates

Flotillin-2 knockout mice were generated and housed as described in^23^. Mouse skin samples were taken from the lateral left side during the dissection of adult wildtype and flotillin-2 knockout mice. Per sample, approximately 100 mg of skin was taken, frozen in liquid nitrogen and homogenised in 500 μl lysis buffer (see cell lysis) supplemented with protease inhibitor cocktail. Tissue homogenisation was done using a tissue lyser (Retsch, Haan, Germany) with three cycles of 2 min at 25 Hz. The samples were further lysed on ice for 45 min and homogenised again with the tissue lyser twice for 2 min. The resulting lysates were cleared by centrifugation. The protein concentration was measured and equal protein amounts were analysed by SDS-PAGE and Western blot.

### Immunofluorescence

Tissue samples were obtained and experimental procedures were performed according to the guidelines of the charitable state controlled foundation Human Tissue & Cell Research, with the written informed patient’s consent. Immunofluorescence was performed with cryosections (5 μm) of normal human skin tissue samples. The cryosections were blocked and permeabilised with 1% BSA and 0.1% digitonin (50 mg/ml in DMSO) for 30 min at room temperature, incubated overnight at 4 °C with the primary antibody (in 1% BSA, 0.1% digitonin), and then incubated for 1 h at room temperature with the secondary antibody and mounted in Gel Mount reagent. Mouse skin samples were frozen in liquid nitrogen in Tissue Tek Compound (Sakura, Tokyo, Japan) and stored at −80 °C. 10 μM cryo-sections were produced, immobilised on glass slides and fixed with cold methanol at −20 °C for 10 minutes. Samples were blocked with 10% mouse serum in PBS for 10 min. The primary antibodies were diluted in 1% mouse serum in PBS and incubated over night at 4 °C. Incubation with the secondary antibodies as well as DAPI staining of the nuclei was performed at RT for 1 h.

Human cell lines were seeded on glass coverslips and cultured for at least 3 days. Cells were treated with either PV IgG/control antibodies or inhibitors (as described below) before fixation, or directly fixed with cold methanol for 5 min at −20 °C. The fixed cells were rinsed with PBS and blocked for 15 min with 1% BSA in PBS at room temperature. Primary and secondary antibody stainings were carried out for 1 h at room temperature in PBS/1% BSA. All samples were analysed with a Zeiss LSM710 Confocal Laser Scanning Microscope (Carl Zeiss, Oberkochen, Germany).

### Bacterial protein expression and purification

All GST and MBP fusion proteins were expressed in the *E.coli* strain Rosetta (De3) pLysS (Novagen). The expression of all Dsg fusion proteins was induced with 0.5–1 mM isopropyl-thiogalactopyranoside (IPTG) for 4–5 h at 37 °C. The expression of the flotillin-GST proteins was induced with 0.15 mM IPTG for 20 h at 20 °C. Bacterial lysis and purification of GST or MBP proteins with glutathione sepharose or amylose resin was done as described earlier^16^.

### GST pulldown

HaCaT cells were lysed for 20 min on ice in GST pulldown lysis buffer (10 mM Tris-HCl, pH 8.0; 150 mM NaCl; 5 mM EDTA; 0.5% Triton X-100; 60 mM N-octylglucoside) supplemented with protease inhibitor cocktail. The lysates were cleared by centrifugation and incubated with 5 mg of GST-tagged proteins or GST, immobilised on glutathione sepharose overnight at 4 °C. The sepharose was washed four times with 1 ml lysis buffer, resuspended in sample loading buffer and boiled for 5 min at 94 °C. Bound proteins were separated by SDS-PAGE and detected by Western blot.

### Pulldown using purified GST and MBP tagged proteins

GST and flotillin-GST proteins were eluted from glutathione sepharose in elution buffer (50 mM Tris-HCl pH 8.0; 150 mM NaCl; 0.1% Triton X-100; 1 mM DTT) containing 40 mM reduced glutathione and protease inhibitor cocktail for 2 h at 4 °C. Eluted proteins were captured by centrifugation and immediately used for direct pulldown experiments. The eluted GST proteins were incubated with purified recombinant MBP or Dsg3-MBP proteins immobilised on amylose resin, in direct pulldown buffer (50 mM Tris-HCl pH 7.5; 150 mM NaCl; 0.01% Triton X-100; 1 mM DTT; 1 mM EDTA; 1% BSA) on ice for 2–3 h. Afterwards, the resin was washed 3 times in direct pulldown buffer, resuspended in sample loading buffer and cooked for 5 min at 94 °C. Proteins were separated by SDS-PAGE and detected by Western blot.

### Dispase based dissociation assay

Dispase based dissociation assays were performed as described in^46^,^47^. Cells were seeded in 12 well plates and grown until confluent. After washing twice with PBS, 1.5 U/ml Dispase II was added and the cells were incubated at 37 °C for 30 to 40 min or until the monolayers detached from the plastic surface. After washing in PBS containing 0.5 mM MgCl_2_ and 0.5 mM CaCl_2_, mechanical stress was applied to the monolayers by pipetting up and down 5 times with a 1 ml pipette. The fragments were fixed with paraformaldehyde and stained with crystal violet. Photographs were taken, and the fragments were counted automatically with the help of the ImageJ software.

### Sequential detergent extraction and isolation of lipid rafts

Sequential detergent extraction was done according to^28^. HaCaT cells were grown until confluent, washed with cold PBS and incubated with 200 μl Triton buffer (1% Triton X-100; 10 mM Tris-HCl, pH 7.5; 140 mM NaCl; 5 mM EDTA; 2 mM EGTA; 1 mM PMSF; 1 μg/ml Leupeptin; 1 μg/ml Pepstatin A) for 10 min on ice. Cells were scraped, vortexed for 30 sec and centrifuged at 14,000 × g for 30 min. The supernatant containing the Triton-soluble proteins was collected. The Triton insoluble proteins (pellet) were subsequently extracted with 400 μl of an SDS/urea buffer (1% SDS; 8 M urea; 10 mM TrisHCl, pH 7.5; 140 mM NaCl; 5 mM EDTA; 2 mM EGTA). 15 μl samples were analysed by SDS-PAGE and Western blot. Stable flotillin knockdown HaCaT cells and control shRNA cells were seeded in equal cell numbers, cultured until confluent, and membrane rafts were isolated according to^48^.

### PV IgG and inhibitor treatment of HaCaT cells

HaCaT cells were serum-starved for 16 h before treatment with 150 μg/ml purified IgG from *pemphigus vulgaris* patients or control-IgG (from a healthy person).

To inhibit Dsg3 endocytosis, HaCaT cells were treated with the inhibitors Dynasore (Sigma-Aldrich) or MitMAB (Abcam) as described in^31^. In brief: Cells were serum-starved overnight and then treated with 80 μM Dynasore for 2 h at 37 °C or with DMSO as a control. Alternatively, the cells were incubated with 30 μM MitMAB for 30 min. BafA is a V-type H(+)-ATPase inhibitor that increases the pH of lysosomes, preventing lysosomal degradation of proteins. The cells were incubated with 50 nM BafA for 24 h at 37 °C and then processed further.

### Statistics

Unless otherwise stated, all experiments were performed at least three times. For the statistical analysis, Western blot bands of proteins were quantified by scanning densitometry using Quantity One Soft-ware (Bio-Rad) and normalised against GAPDH. Data are shown as the mean ± SD. Statistical comparisons were made using Student’s t test, one-way analysis of variance (ANOVA) or two-way ANOVA with Bonferroni’s multiple comparison test, as appropriate, using GraphPad Prism 5 software. Values of p < 0.05 were considered significant (*), whereas values of p < 0.01 and p < 0.001 were defined very significant (**) and highly significant (***), respectively.

### Electronic manipulation of images

The fluorescence images have in some cases as a whole been subjected to contrast or brightness adjustments. No other manipulations have been performed unless otherwise stated.

### Ethical statement

Ethical approval was not required for this study because under the German Animal Welfare Act, a procedure that only involves the euthanasia of the animal and post-mortem retrieval of organs is not subject to a special ethical approval. All procedures with laboratory animals used in this study have been declared to the Animal Welfare Office of the Justus-Liebig-University Giessen (registry number 475_M). The mice were treated in strict accordance with the recommendations of the Guide for the Care and Use of Laboratory Animals of the National Institutes of Health and of local authorities. All animals were sacrificed after deep isofluran anesthesia to ensure minimal suffering. Human tissue samples were obtained and the experimental procedures were performed according to the guidelines of the charitable state controlled foundation Human Tissue & Cell Research and the Declaration of Helsinki, with a written informed patients’ consent obtained by the foundation, as stated in^49^.

## Additional Information

**How to cite this article**: Völlner, F. *et al*. Loss of flotillin expression results in weakened desmosomal adhesion and *Pemphigus vulgaris*-like localisation of desmoglein-3 in human keratinocytes. *Sci. Rep.* **6**, 28820; doi: 10.1038/srep28820 (2016).

## Supplementary Material

Supplementary Information
