# Supplementary material for: Loss of flotillin expression results in weakened desmosomal adhesion and Pemphigus vulgaris-like localisation of desmoglein-3 in human keratinocytes
Source: Sci Rep. 2016 Jun 27;6:28820. doi: 10.1038/srep28820 (PMC4922016; doi:10.1038/srep28820)
Supplement: Supplementary Information [file srep28820-s1.pdf]

Supplementary Figures to Manuscript:

Loss of flotillin expression results in weakened desmosomal adhesion and *Pemphigus vulgaris*-like localization of desmoglein-3 in human keratinocytes

Frauke Völlner, Jawahir Ali, Nina Kurrle, Yvonne Exner, Rüdiger Eming, Michael Hertl, Antje Banning, Ritva Tikkanen*

Supplementary Figure S1, Völlner et al.

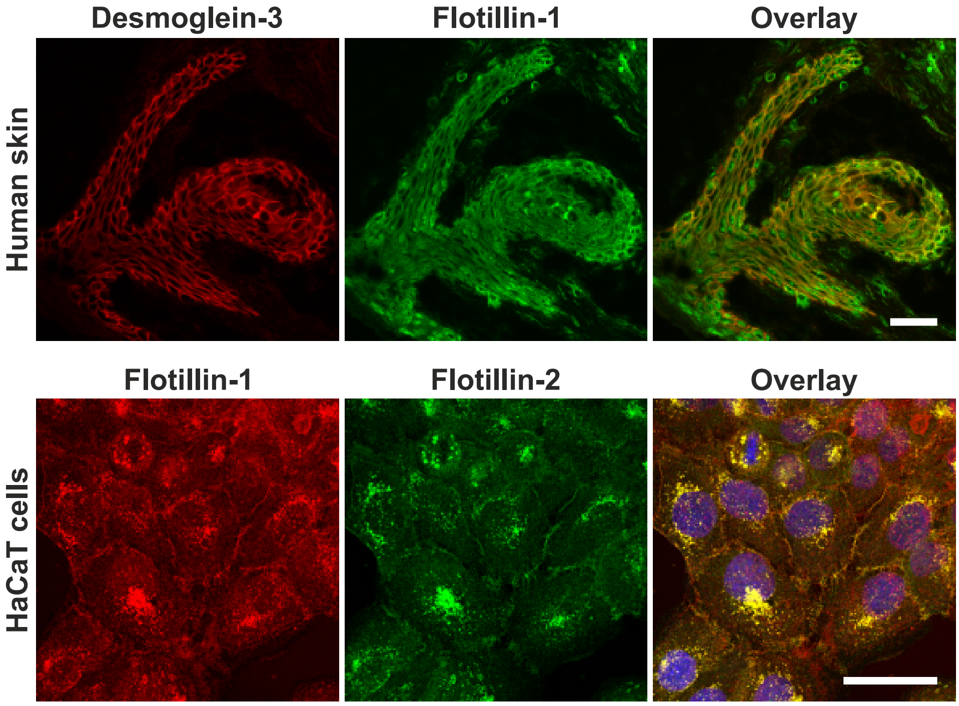

Supplementary Figure S1. Desmoglein-3 and flotillin-1 colocalise in human epidermis. Human skin cryosections were immunostained for Dsg3 (red) and flotillin-1 (green). In human epidermis, flotillin-1 is expressed throughout the epidermal layer and colocalises with Dsg3 in keratinocytes of the stratum basale and suprabasale. In HaCaT keratinocytes, flotillin-1 and flotillin-2 extensively colocalise at the cell membrane and in intracellular vesicular structures.

Supplementary Figure S2, Völlner et al.

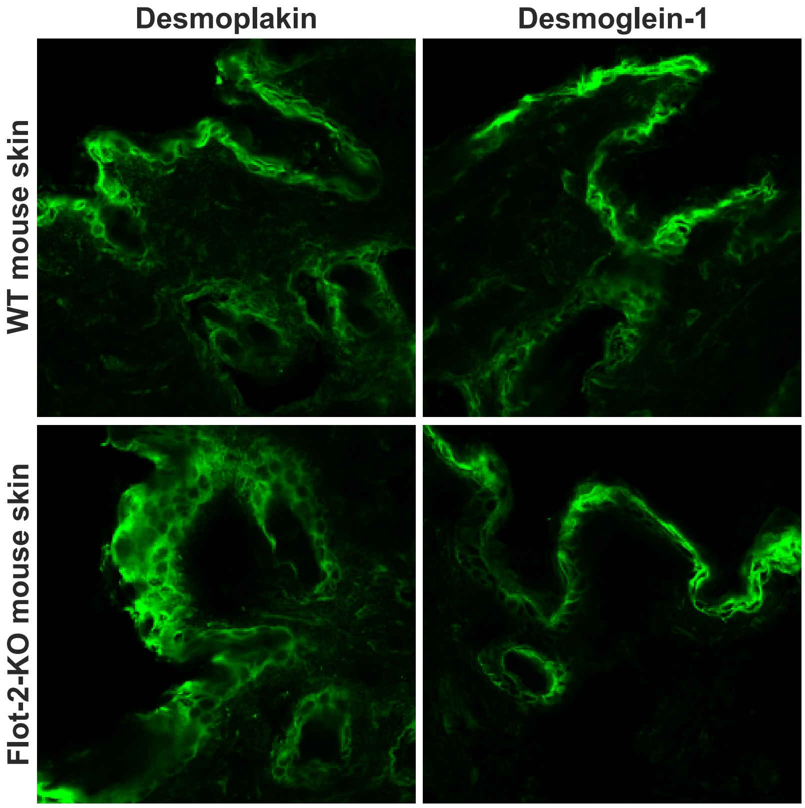

Supplementary Figure S2. Staining of desmoplakin and Desmoglein-1 in mouse epidermis. Mouse skin cryosections from control and flotillin-2 knockout mice were immunostained for desmoplakin or Dsg1 (green). No major difference in the staining patterns could be observed between control and knockout mice.

Supplementary Figure S3, Völlner et al.

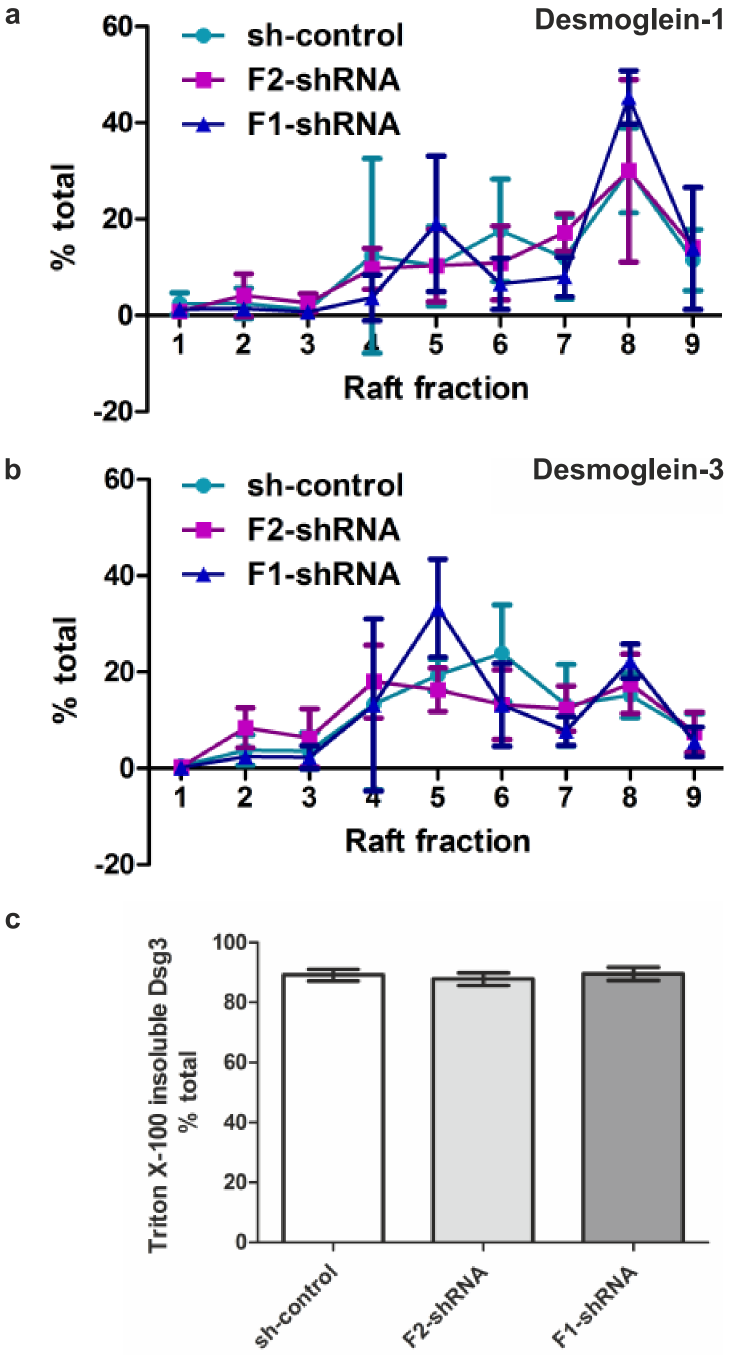

Supplementary Figure S3. Flotillin knockdown does not affect the raft association or detergent solubility of Desmoglein-1 and Desmoglein-3. (a-b) Quantification of the data shown in Figure 5. No significant differences in the percentage of Dsg1 or Dsg3 associated with the raft fractions could be seen between control and flotillin knockdown HaCaT cells. (Statistical analysis by two-way ANOVA) (c) The percentage of Triton X-100 soluble Dsg3 is not significantly altered upon flotillin knockdown.

Supplementary Figure S4, Völlner et al.

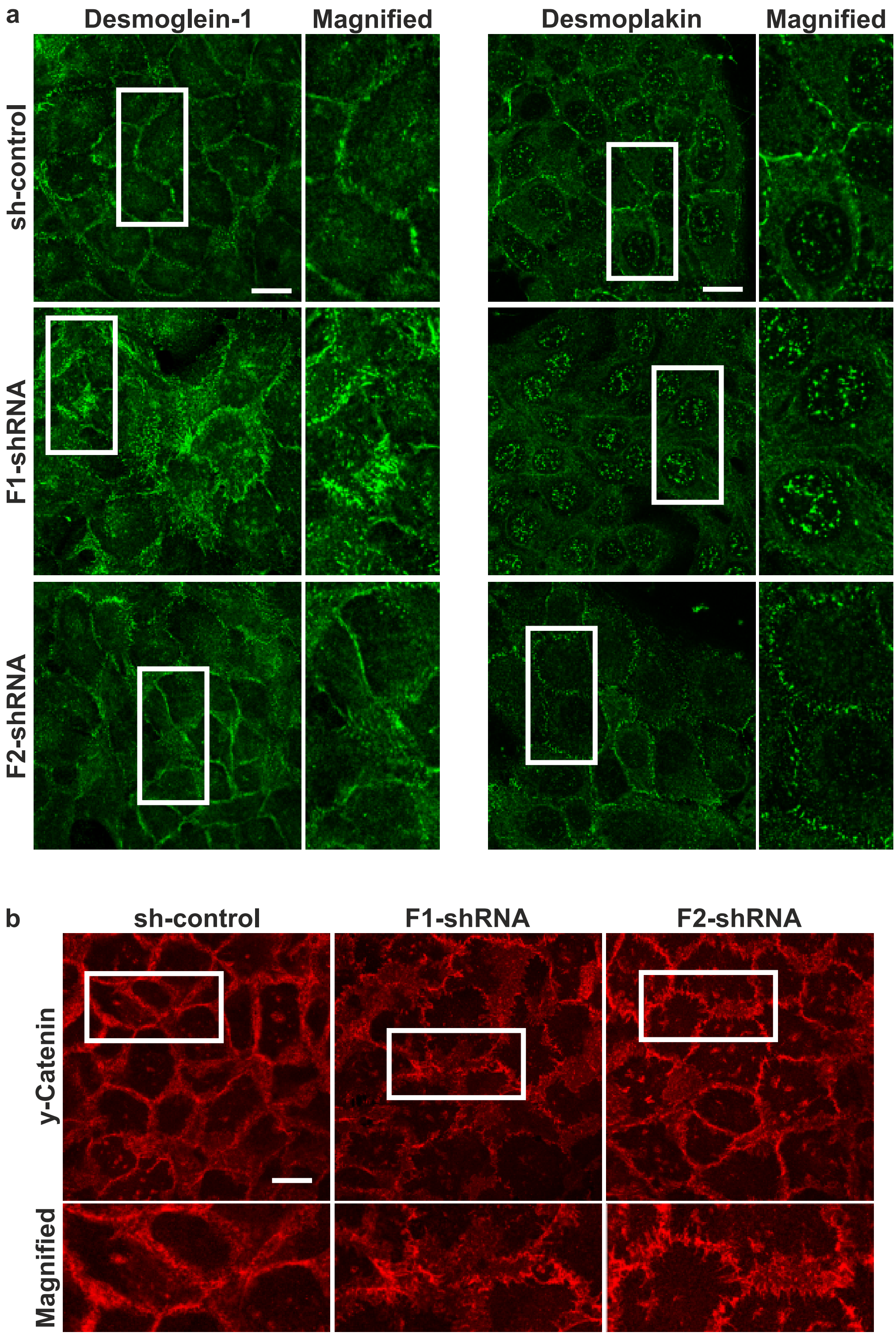

Supplementary Figure S4. Loss of flotillins results in altered localisation of desmosomal proteins in HaCaT keratinocytes. HaCaT cells depleted of flotillin-1 (F1-shRNA), flotillin-2 (F2-shRNA) or control shRNA cells (sh-control) were grown for three days on glass coverslips, fixed and stained with (a) Dsg1 (left) or desmoplakin (right) or (c) γ-catenin antibodies. Scale bar: 20 μm.

Supplementary Figure S5, Völlner et al.

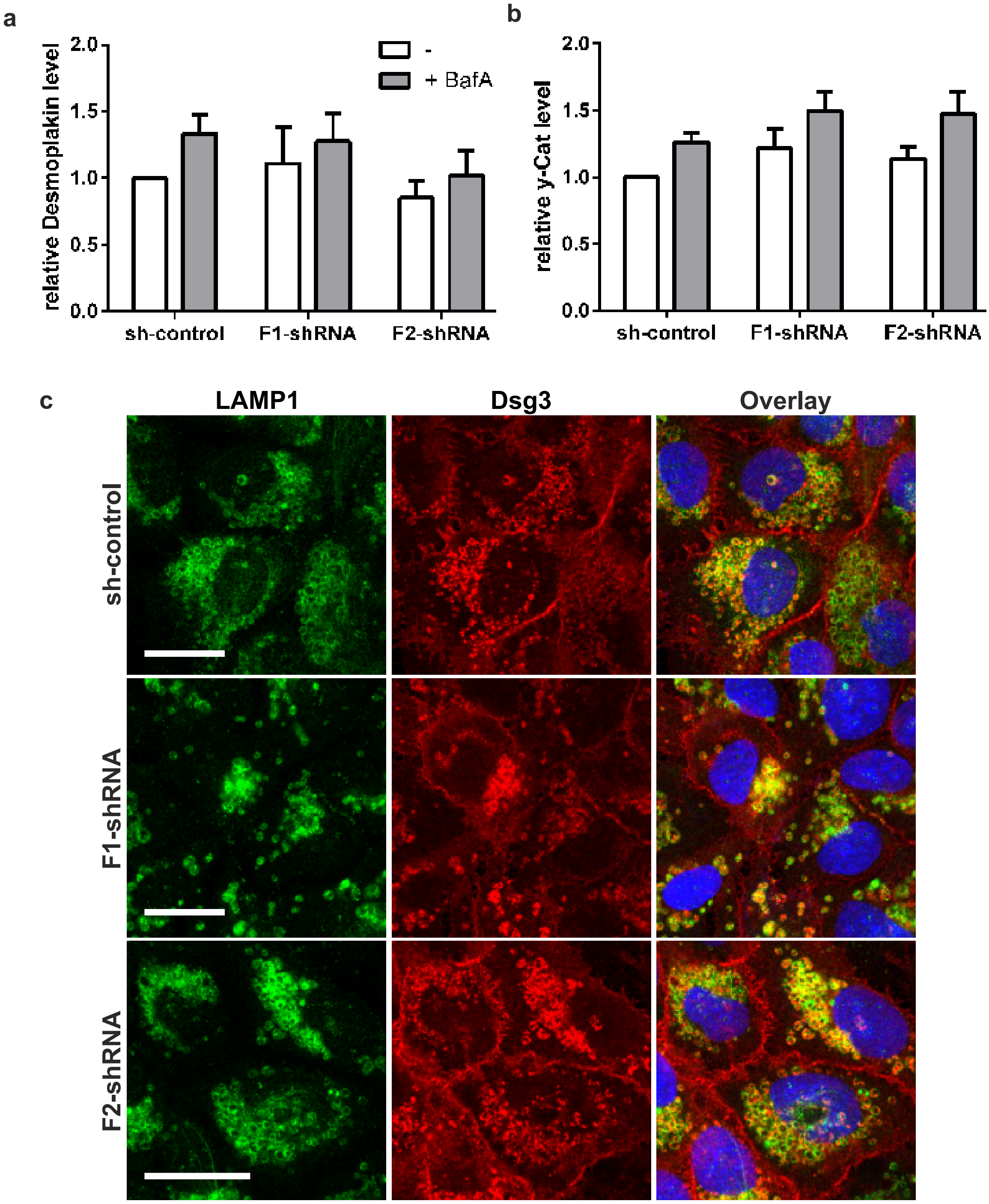

Supplementary Figure S5. Inhibition of lysosomal degradation or endocytosis does not change desmoplakin and γ-catenin levels but results in lysosomal accumulation of Dsg3 in flotillin knockdown cells. Flotillin depleted or control HaCaT cells were treated for 24 h with BafA (50 nM) to block lysosomal degradation. (a-b) Quantification of data shown in Fig. 7. No significant differences could be detected. Cell lysates were analysed by Western blot, quantified by scanning densitometry and normalised against GAPDH. Bars represent the mean ± SD of 6 independent experiments. Two-way ANOVA. (c) After BafA treatment, Dsg3 (red) accumulates in lysosomes and colocalises with the lysosomal marker LAMP1 (green). Scale bar: 20 µm.

Supplementary Figure S6, Völlner et al.

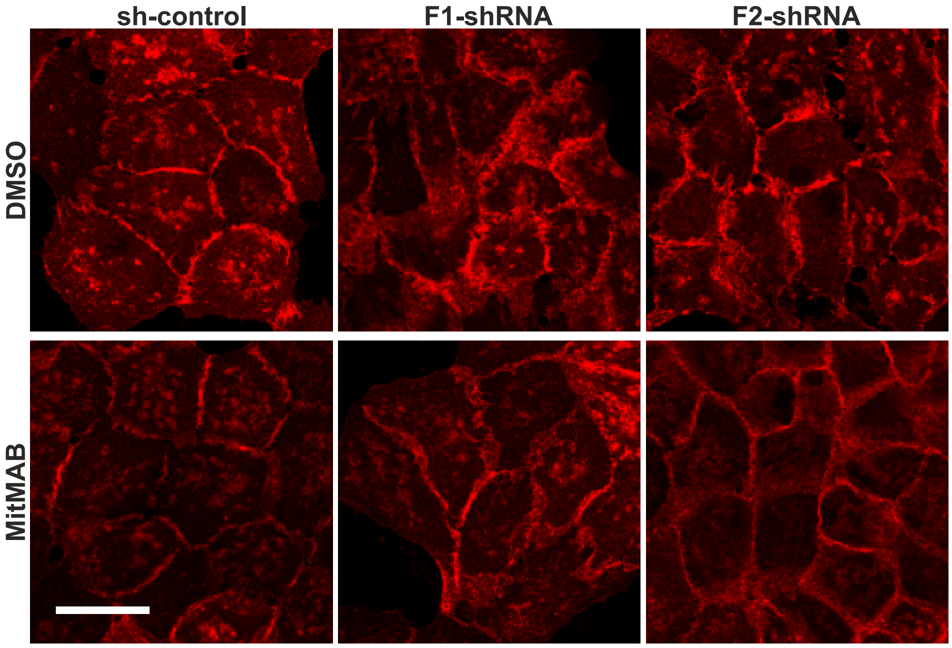

Supplementary Figure S6. Inhibition of dynamin-dependent endocytosis improves desmosomal morphology in flotillin knockdown HaCaT cells. Flotillin knockdown and control cells were serum-starved overnight and treated for 30 min with 30 μM MitMAB to inhibit Dynamin-dependent endocytic uptake of Dsg3 from the plasma membrane. Cells were fixed and immunostained for Dsg3. Scale bar: 20 μm.
